# Synaptic Organization of the Human Temporal Lobe Neocortex as Revealed by High-Resolution Transmission, Focused Ion Beam Scanning, and Electron Microscopic Tomography

**DOI:** 10.3390/ijms21155558

**Published:** 2020-08-03

**Authors:** Astrid Rollenhagen, Bernd Walkenfort, Rachida Yakoubi, Sarah A. Klauke, Sandra F. Schmuhl-Giesen, Jacqueline Heinen-Weiler, Sylvia Voortmann, Brigitte Marshallsay, Tayfun Palaz, Ulrike Holz, Mike Hasenberg, Joachim H.R. Lübke

**Affiliations:** 1 Institute of Neuroscience and Medicine INM-10, Research Centre Jülich GmbH, Leo-Brandt Str., 52425 Jülich, Germany; a.rollenhagen@fz-juelich.de (A.R.); r.yakoubi@fz-juelich.de (R.Y.); sandra.giesen@rwth-aachen.de (S.F.S.-G.); b.marshallsay@fz-juelich.de (B.M.); t.palaz@fz-juelich.de (T.P.); u.holz@fz-juelich.de (U.H.); 2 Imaging Center Essen (IMCES), Electron Microscopy Unit (EMU), Medical Faculty of the University of Duisburg-Essen, Hufelandstr. 55, 45147 Essen, Germany; bernd.walkenfort@uk-essen.de (B.W.); sarah.klauke@stud.uni-due.de (S.A.K.); jacqueline.heinen-weiler@uk-essen.de (J.H.-W.); sylvia.voortmann@uk-essen.de (S.V.); 3 Department of Psychiatry, Psychotherapy and Psychosomatics, Medical Faculty/RWTH University Hospital Aachen, Pauwelsstr. 30, 52074 Aachen, Germany; 4 JARA Translational Brain Medicine, Jülich/Aachen, Germany

**Keywords:** human temporal lobe neocortex, synaptic boutons, active zones, synaptic vesicles, transmission and focused ion beam scanning EM, EM tomography, quantitative three-dimensional models of synaptic boutons

## Abstract

Modern electron microscopy (EM) such as fine-scale transmission EM, focused ion beam scanning EM, and EM tomography have enormously improved our knowledge about the synaptic organization of the normal, developmental, and pathologically altered brain. In contrast to various animal species, comparably little is known about these structures in the human brain. Non-epileptic neocortical access tissue from epilepsy surgery was used to generate quantitative 3D models of synapses. Beside the overall geometry, the number, size, and shape of active zones and of the three functionally defined pools of synaptic vesicles representing morphological correlates for synaptic transmission and plasticity were quantified. EM tomography further allowed new insights in the morphological organization and size of the functionally defined readily releasable pool. Beside similarities, human synaptic boutons, although comparably small (approximately 5 µm), differed substantially in several structural parameters, such as the shape and size of active zones, which were on average 2 to 3-fold larger than in experimental animals. The total pool of synaptic vesicles exceeded that in experimental animals by approximately 2 to 3-fold, in particular the readily releasable and recycling pool by approximately 2 to 5-fold, although these pools seemed to be layer-specifically organized. Taken together, synaptic boutons in the human temporal lobe neocortex represent unique entities perfectly adapted to the “job” they have to fulfill in the circuitry in which they are embedded. Furthermore, the quantitative 3D models of synaptic boutons are useful to explain and even predict the functional properties of synaptic connections in the human neocortex.

## 1. Introduction

One major and important question in synaptic neuroscience is whether findings about the structural compositions of the adult, developmental, and pathologically altered brain in experimental animals can be transferred one to one to the human brain. This can be partially attributed to the non-availability of ultrastructural well-preserved human tissue samples that allow investigations at the fine-scale high-resolution cellular and subcellular electron microscopy (EM) level. However, post-mortem human brains from deceased patients are more frequently available from body donations and were hence used in meanwhile numerous structural studies. For example, to further verify brain regions in humans [1,2,3,4], structural investigation used the Golgi-impregnations [5,6,7] or intracellular Lucifer yellow injections in fixed brain slices [8,9,10], and even molecular investigation about receptor architectonics [11,12]. However, structural investigations at the cellular and subcellular EM level are limited by the poor ultrastructural preservation of tissue samples obtained from post-mortem brains. This can be mainly attributed to hypoxia-mediated autolysis within the long time window between the decease of the patient and the removal of the brain by the pathologist.

In the late years of the last century, patients undergoing epilepsy or tumor brain surgery donated tissue samples of the human brain under strong ethical permission. Hence, it became possible to investigate structural and functional aspects of synaptic transmission and plasticity in the human brain in acute brain slice preparations [13,14,15,16,17,18,19].

However, studies about the synaptic organization of the neocortex and hippocampus, particularly coherent quantitative structural investigations, are still rare, particularly from the presynaptic terminal [20,21,22,23,24,25].

The human temporal lobe neocortex (TLN) is ideally suited for such investigations since it has to be removed to access the hippocampus proper during epilepsy surgery. Furthermore, the TLN is of importance because it represents a highly specialized associative, homotypic granular, six-layered neocortex involved in auditory, visual, vestibular, linguistic, and olfactory processing that is linked to other multimodal associations areas such as the limbic and various other sensory systems. Thus, the TLN occupies about approximately 20% of the total volume of the human cerebral cortex [26]. Hence, the TLN is regarded as a higher-order, but not primary, or early sensory neocortex [27]. Finally, the growing interest in working on the TLN is based on its involvement in several neurological diseases, most importantly as the area of origin and onset for the most common form of epilepsy, the temporal lobe epilepsy [28,29]. In summary, the TLN represents an important region in the normal and pathologically altered human brain. 

The aim of the present study was to demonstrate that high-end, high-resolution transmission EM (TEM), focused ion beam scanning EM (FIB-SEM), and EM tomography are useful tools to investigate the synaptic organization of the human brain, exemplified by non-epileptic access tissue samples of the human TLN from patients that underwent epilepsy surgery. Here, we demonstrate that the structural preservation of brain tissue samples taken and fixed immediately after tissue removal was extremely well preserved, thus leading to reliable and reproducible results in the investigation and quantification of the neuronal and synaptic organization of the normal and pathologically altered human neocortex.

Beside similarities, human synaptic boutons (SBs) differ substantially in several structural parameters from their counterparts in experimental animals. In particular, the shape and size of active zones (AZs), the structural equivalent of a functional neurotransmitter release site, and the three functionally defined pools of synaptic vesicles (SVs), namely the readily releasable (RRP), the recycling (RP), and resting pool were strikingly different. Thus, SBs in the human TLN represent unique entities, and their detailed ultrastructural characterization will help very much to improve our understanding about these structures under healthy and pathological conditions.

## 2. Results

The main goal of our investigations was and still is to describe the synaptic organization of a cortical column, layer by layer, exemplified for the TLN in the normal [24,25] and pathologically altered brain (work in progress). It has to be noted here that a thorough and detailed quantitative analysis will be the subject of separate publications in addition to already released data [24,25]. With the present manuscript, we want to give a more general view of the synaptic organization of the human TLN and to demonstrate the possibility to generate quantitative 3D models of SBs and their target structure using both TEM and FIB-SEM datasets.

### 2.1. Comparison of TEM and FIB-SEM to Investigate the Neuropil and Synaptic Organization of the TLN

**Figure 1:**
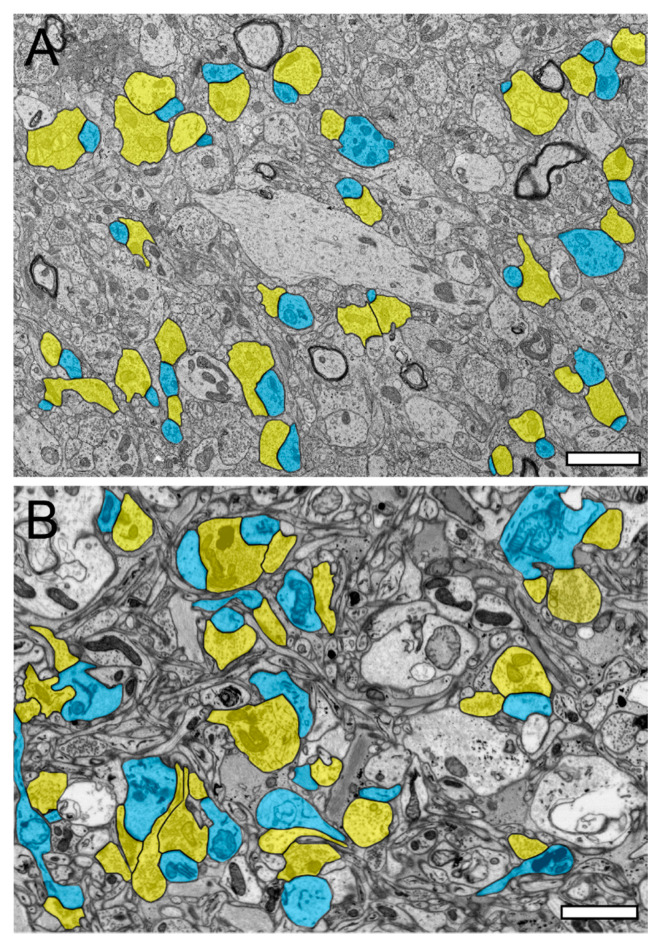
Synaptic organization of the neuropil in the human temporal lobe neocortex (TLN). (**A**), Low-power electron microscopy (EM) micrograph of the neuropil of L2 in the gyrus temporalis medialis of the TLN processed for TEM analysis. Here, several synaptic complexes, composed of a dendritic shaft or spine and a synaptic bouton, are highlighted in transparent yellow (synaptic boutons, or SBs) and transparent blue (dendritic shafts or spines). Note the high density of axo-spinous synaptic complexes. (**B**), Same layer, gyrus, and color code as shown in (**A**), but here, the tissue sample was processed for focused ion beam scanning EM (FIB-SEM) analysis. Note the different structural appearance of the neuropil between the TEM (**A**) and FIB-SEM (**B**) processed tissue samples. Scale bar in (**A**,**B**), 1 µm.

**Figure 2:**
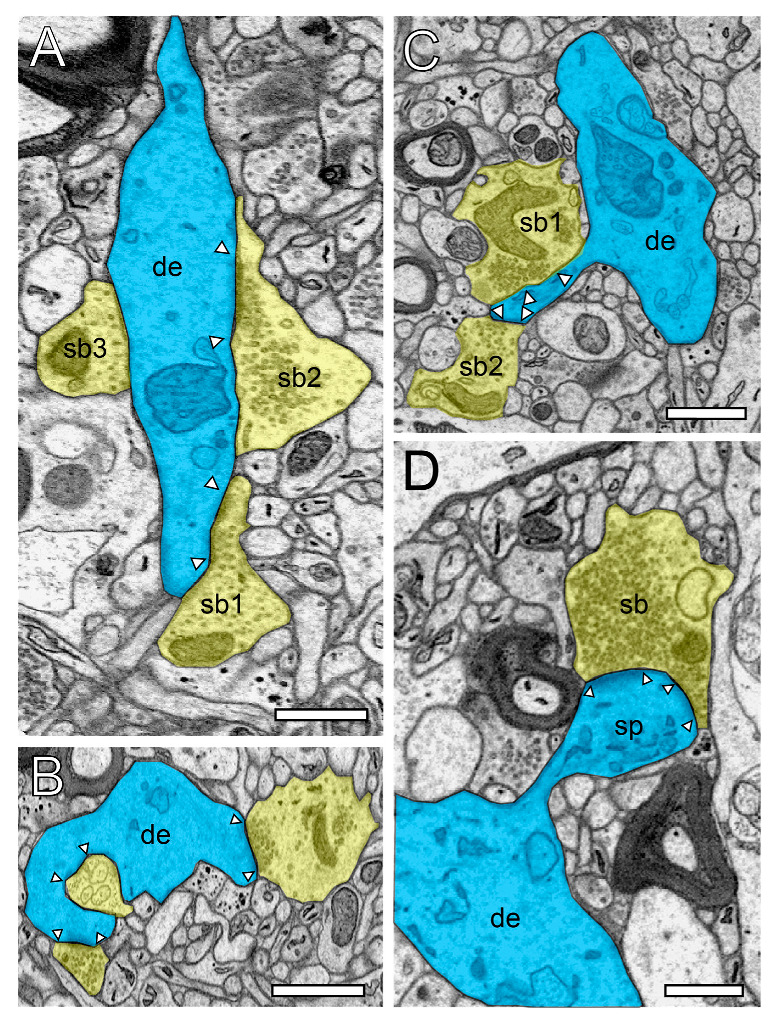
Structural characteristics of SBs and their target structures in the human TLN as revealed by FIB-SEM. (**A**), Large dendrite (de, transparent blue) with three terminating SBs (sp1–sp3, transparent yellow of different shape and size in L6 of the gyrus temporalis medialis. In two SBs (sb1, sb2), a clear AZ is visible, whereas sb3 is lacking an AZ. (**B**), Small caliber dendrite (de) receiving synaptic input from three SBs, one of which invaginating the dendrite in L5 of the gyrus temporalis medialis. (**C**), Elongated spine (sp) emerging with a long spine neck from a dendrite (de) in L3 of the gyrus temporalis medialis with two opposing SBs (sb1, sb2) terminating on the spine head. AZs are marked by arrowheads. Same color code as in A. (**D**), Large mushroom spine (sp) with a short spine neck originating from a dendritic segment (de) in L2 of the gyrus temporalis inferior receiving input from a relatively large end terminal SB with synaptic vesicles (SVs) distributed over the entire terminal. In all images, AZs are marked by arrowheads, and the same color code is used as in (**A**). Scale bar in (**A**–**D**), 0.5 µm.

**Figure 3:**
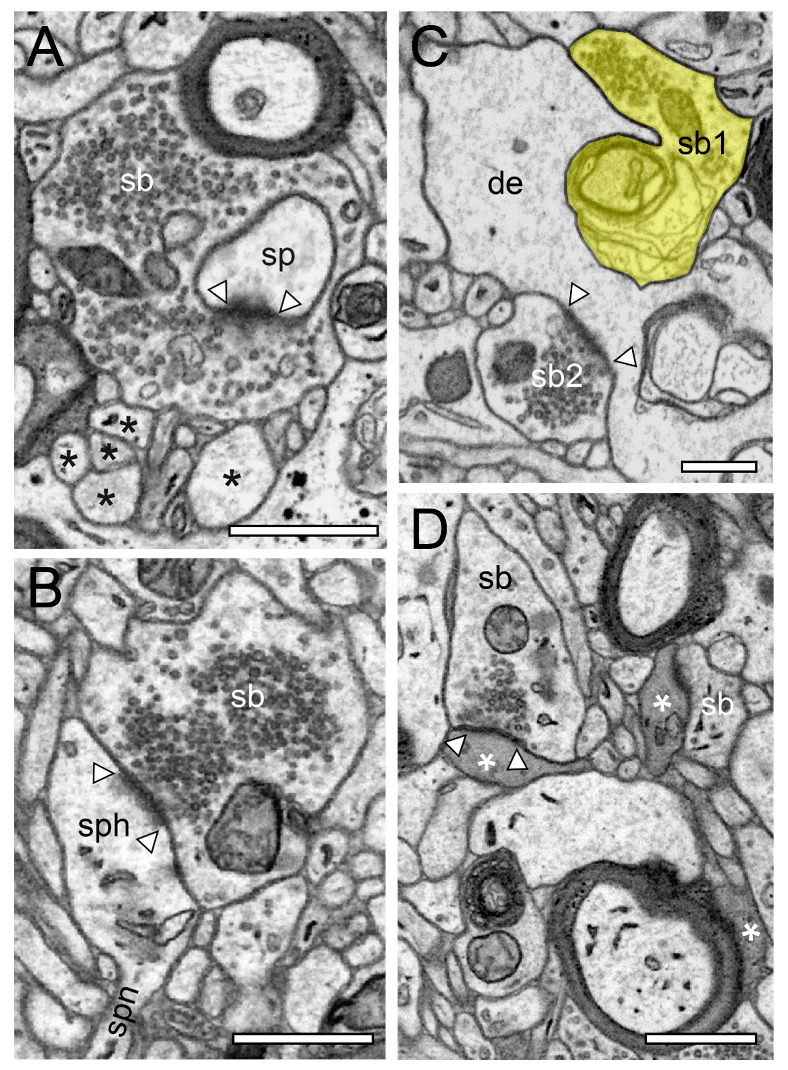
Structural characteristics of SBs in the human TLN as revealed by FIB-SEM. (**A**), Large SB (sb) invaginating a dendritic spine (sp) in L5 of the gyrus temporalis medialis. The AZ is marked by arrowheads. Note the cluster of unmyelinated axons (asterisks) close to the end terminal bouton. (**B**), Large synaptic bouton (sb) terminating on a large spine head (sph) of a short-necked spine (spn) in L6 of the gyrus temporalis. (**C**), Dendrite (de) with two SBs (sb1, sb2); sb1 with a large tangled structure (highlighted in transparent yellow) within the interior of the bouton invaginating the dendrite, whereas sp2 shows a “normal” appearance. The AZ in sb2 is marked by arrowheads. (**D**), Small caliber astrocytic processes (asterisks) identifiable by their darker appearance in the surrounding neuropil in L6 of the gyrus temporalis inferior. Infrequently, these astrocytic processes receive synaptic input by an SB (sb) identifiable by the establishment of a prominent AZ (arrowheads). Scale bar in (**A**–**D**), 0.5 µm showed layer-specific differences. It has been shown recently that the size of the readily releasable (RRP) dynamically regulates multivesicular release in mice [30], which also seemed to be the case at SBs in the human TLN.

**Figure 4:**
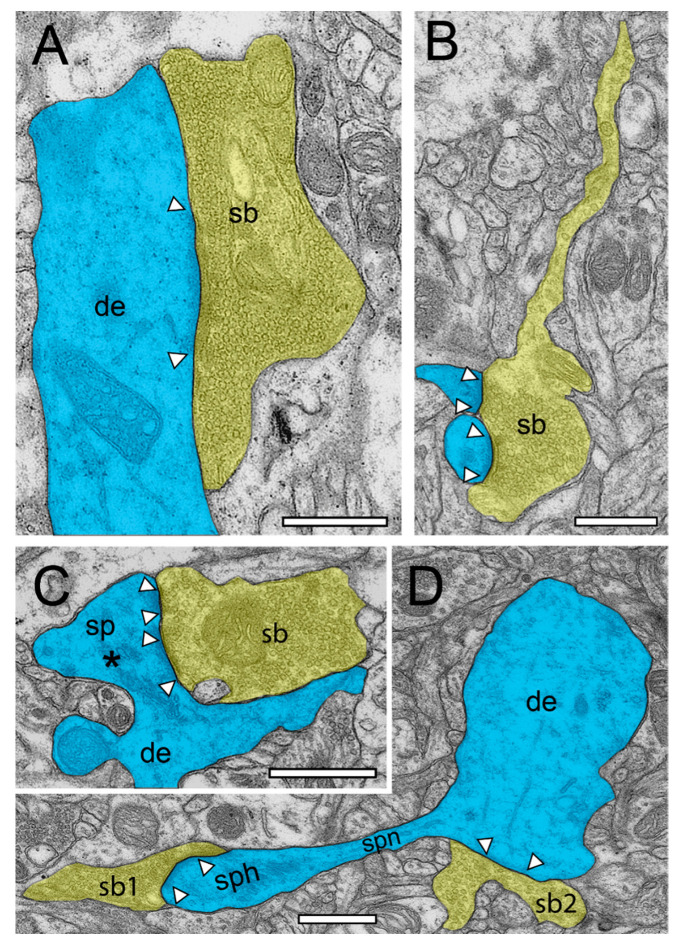
Structural characteristics of SBs and their target structures in the human TLN as revealed by TEM. (**A**), Large putative inhibitory (sb, transparent yellow) terminating on a large dendrite (de, transparent blue) in L2 of the gyrus temporalis inferior with a large macular, non-perforated AZ (arrowheads) containing thousands of SVs. (**B**), Typical example of an axonal segment, giving rise to an end terminal SB (sb) innervating two adjacent small dendritic spines with relatively small AZs (arrowheads) in L3 of the gyrus temporalis inferior. Note the appearance of SVs also in the axon. Same color code as in A. (**C**), End terminal SB (sb) synapsing on a large stubby spine (sp) with a large spine head emerging directly from a dendrite (de) in L6 of the gyrus temporalis medialis. Note the two AZs (arrowheads) and the large spine apparatus (asterisk) in the spine head. Same color code as in (**A**). (**D**), A small dendritic segment (de) giving rise to an elongated spine with a small spine neck (spn) leading to a large spine head (sph). Two SBs (sb1, sb2) terminate directly on the spine head (sb1) and on the dendrite (sb2). AZs are marked by arrowheads. Same color code as in (**A**). Scale bar in (**A**–**D**), 0.5 µm.

**Figure 5:**
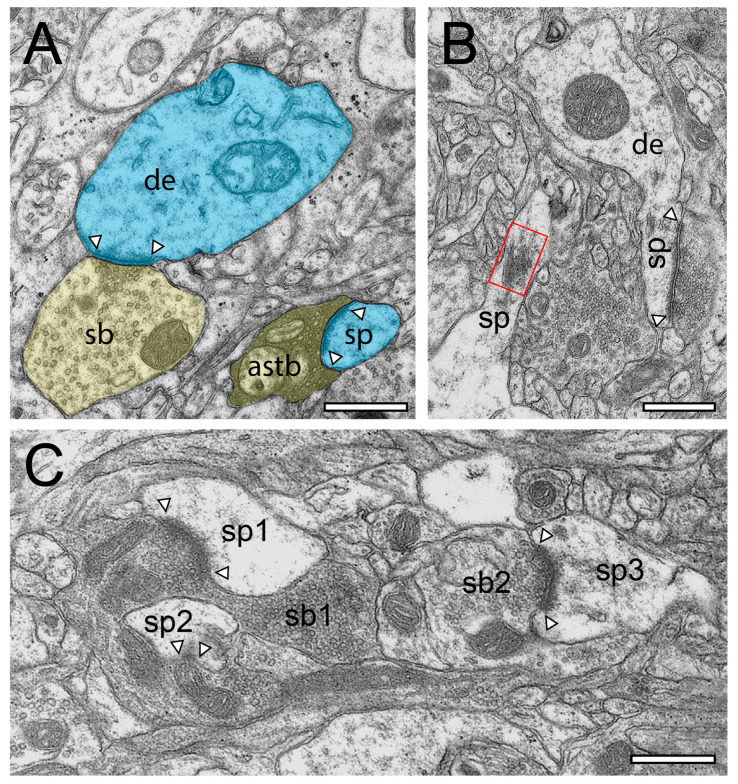
Structural characteristics of SBs in the human TLN as revealed by TEM. (**A**), EM micrograph of SBs (sb, transparent yellow) terminating on a dendrite (de, transparent blue) with an adjacent “astrocytic bouton” (astb, transparent yellow) terminating on a small dendritic spine (transparent blue) in L3 of the gyrus temporalis medialis. Note the dark appearance typical for astrocytes and the content of vesicles containing gliotransmitter. AZs are marked by arrowheads. (**B**), two spines (sp) with a relatively large spine head and short spine necks one containing a prominent spine apparatus (framed area) in L6 of the gyrus temporalis medialis. The right spine with a large macular, non-perforated AZ (arrowheads) receiving input from an end terminal SB, whereas on the left spine display, no obvious contact is visible. (**C**), Large SBs (sb1) invaginating two spines (sp1, sp2) in L2 of the gyrus temporalis inferior next to another spine (sp3) receiving input from another SB (sb2). AZs are marked by arrowheads. Scale bar in (**A**–**C**), 0.5 µm.

**Figure 6:**
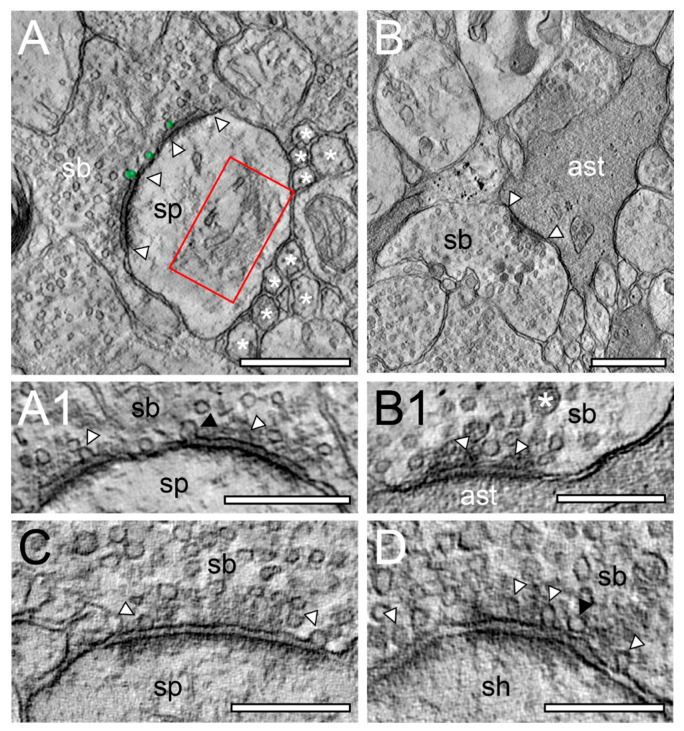
EM tomography of synaptic complexes and active zone with “docked” SVs. (**A**), Single EM micrograph of a tomographic TILT series through a synaptic complex between a spine (sp) and a synaptic bouton (sb) in L6a of the human TLN. The framed area indicates a large spine apparatus and arrowheads indicate the AZ. “Docked” vesicles are highlighted in transparent green. Note the cluster unmyelinated at the synaptic complex. (**A1**), High magnification of the AZ shown in A. The synaptic bouton (sb) is on top the spine (sp) at the bottom of the images. Here, the “docked” SVs (arrowheads) are clearly visible. Note the omega-shaped SV (black arrowhead) indicating the already occurred release of a quantum of neurotransmitter. (**B**), Synaptic bouton (sb) establishing a contact (arrowheads) with an astrocytic process (ast) identified by its darker appearance in L6b of the human TLN. (**B1**), Higher magnification of the AZ shown in B. Here, two SVs are already fused with the presynaptic membrane as marked by arrowheads. Note also the large dense core vesicle (asterisk). (**C**), Large macular, non-perforated AZ between a spine (sp) and a synaptic bouton (sb) with two ‘docked vesicles’ in L5 of the human TLN. (**D**), Large non-perforated AZ at a synaptic complex between a dendritic shaft (sh) and a synaptic bouton (sb) with four “docked” SV (arrowheads), two with an omega-shaped appearance. Note the SV (black arrowhead) close, but not fused with the presynaptic density. Scale bar in (**A**), (**B**) 0.5 µm and in (**A1**), (**B1**), (**C**), (**D**) 0.25 µm.

In the beginning of the presented work, we evaluated if in our hands, TEM or (FIB-) SEM represented the best-suited imaging modality to characterize the organization of the neuropil and its components throughout the different layers and temporal gyri in highest detail. In summary, we found that both methods were able to visualize key ultrastructural sub-elements of the investigated tissue, but with our so far established and refined sample preparation and image acquisition protocols, the contrast in the resulting FIB-SEM images was clearly poorer compared to corresponding TEM images. In detail, this was especially critical for the visualization of biological membranes (compare Figure 1A with Figure 1B, Figure 2 and Figure 3 [FIB-SEM] with Figure 4 and Figure 5 [TEM]). The undoubtful identification of the membrane borders was a prerequisite for the successful segmentation of synaptic key elements such as the AZs and for elucidating the size and shape of SVs. At lower magnifications of around ×6000 to ×8000, the shape of AZs and SVs were clearly visible and distinguishable from each other in the TEM pictures (Figure 1A, Figure 4 and Figure 5) and also visible in the FIB-SEM image material (Figure 1B, Figure 2 and Figure 3; Movie S1). However, at higher magnifications, fine structural elements such as membrane areas of AZs and SVs appeared more blurry in the FIB-SEM material, so that the assessment of distance parameters became more difficult and thus non-precise (compare Figure 2 with Figure 4 and Figure 3 with Figure 5, Movie S1). The blurriness was a result of two predominant factors. First, the real point resolution of the FIB-SEM images was worse in contrast to the TEM data (10,444 × 11,129 pixel; Figure 1A) and second, the signal-to-noise ratio in the FIB-SEM pictures was poorer due to the low contrast and the resulting non-optimal pixel dwell times during image acquisition. The quantitative description of the SVs and their AZ environment was very difficult so that measurements of their numbers, diameters, and distances from the presynaptic density were prone to false interpretation, which had an enormous impact on the definition (measurement) of the RRP and RP. This problem did not occur in the TEM processed material, where SVs could be undoubtfully identified and separated from each other, even at the single vesicle resolution. With that image quality, it was possible to unequivocally sort SVs into the three functionally defined vesicle pools by perimeter measurements (see below) and EM tomography (Figure 6, Movie S1).

**Figure 7:**
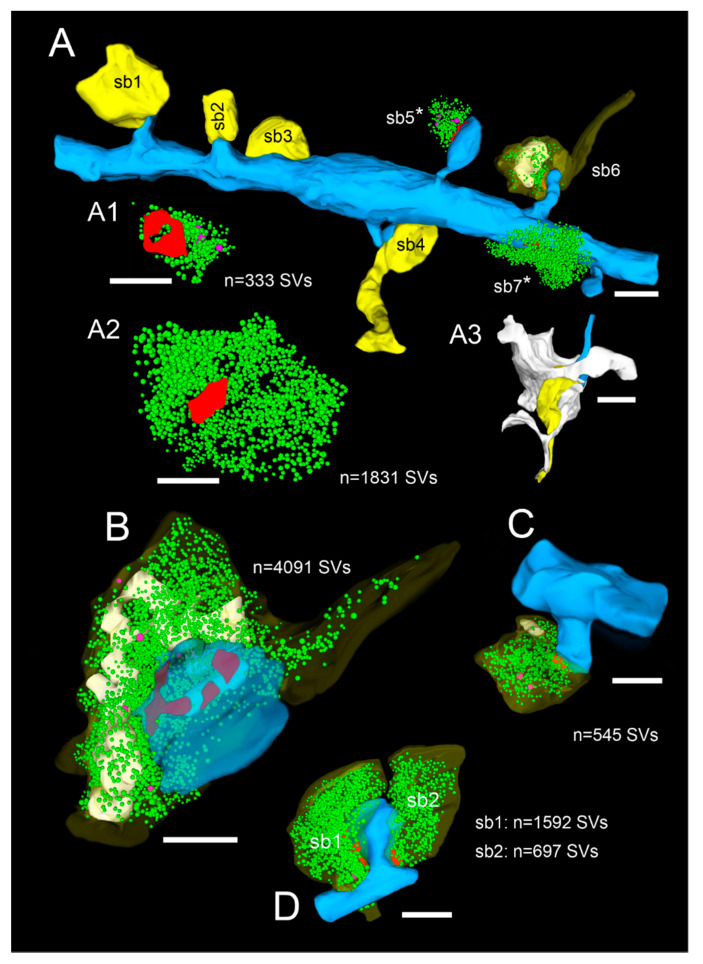
3D-volume reconstructions of SBs and their target structures in the human TLN 3D reconstructed from FIB-SEM and TEM z-stacks. (**A**), Dendritic segment (blue) in L2 receiving dense synaptic input by seven SBs (yellow, sb1–sb7) reconstructed from a large z-stack using FIB-SEM. In two SBs, the bouton cover was omitted, and in one, it was made transparent to allow the visualization of the total pool of SVs (green dots), the AZ (red), and mitochondria (white). Note the different shape and size of the synaptic terminals and the content of SVs. (**A1**), (**A2**), (**3D**)—reconstruction of the total pool of SVs in the spine (sb5) and shaft (sb7) SB shown in A as marked by asterisks. Note the large difference in the pool of SVs and the lack of mitochondria in the large shaft bouton. (**A3**), Representative example of the dense astrocytic ensheathment (white contour) of an axo-spinous synaptic complex. The astrocytic coverage isolates the synaptic complex from the neuropil and other synaptic complexes, and fine astrocytic processes can be followed to reach the synaptic cleft. (**B**–**D**), Three representative examples of SBs and their target structures 3D reconstructed based on serial ultrathin sections and TEM imaging. All SBs are given in their proportional size to each other. (**B**), Large end terminal SB in L2. Here, the cover of the SB and that of the postsynaptic spine was made transparent yellow and blue to visualize the pool of SVs (green dots), mitochondria (white), and the three AZs (red) in the presynaptic terminal. (**C**), SB with a comparably small pool of SVs terminating on a stubby spine in L2. (**D**), Two SBs (sb1, sb2) terminating on the same mushroom spine in L2. Note the different geometry and size of SBs, the different number, shape, and size of the AZs, the pool of SVs, and the content or lack of mitochondria. Scale bar in (**A**–**D**), 0.5 µm.

Nevertheless, a series of 2D images, representing a stack of single-plane data of both EM methodologies, could be used to generate quantitative 3D models of SBs and their target structures in the human TLN (Figure 7). Again, the better 2D image quality of the TEM approach facilitated the segmentation of the image data and therefore offered a significantly better and more reliable 3D reconstruction and interpretation of structural sub-elements, such as that of AZs and SVs.

### 2.2. Very Special Entities: Synaptic Boutons in the Human TLN

One of the major questions in synaptic neuroscience is whether results obtained in experimental animals can be transferred one-to-one to the human brain. As already stated briefly above, research on the human brain was for a long time restricted to post-mortem brains via donations. However, it turned out that this tissue is not suitable for fine-scale, high-resolution TEM and FIB-SEM due to the comparably poor ultrastructural preservation. To overcome this problem, non-epileptic access tissue from epilepsy or brain tumor surgery became an excellent alternative and thus the method of choice for such investigations. 

Using non-epileptic neocortical access tissue taken from epilepsy surgery, we have started to study the layer-specific quantitative synaptic organization of the TLN (Figure 1, Figure 2, Figure 3, Figure 4 and Figure 5, Movie S1). So far, layer (L) 4—the receiving input layer of signals from the sensory periphery, thus representing the first station of intracortical information processing—and L5, the major output layer, was quantitatively analyzed in detail and were already published [24,25]. For the remaining layers L1, L2, L3, and L6, work is in progress.

SBs in the human TLN vary substantially in size ranging from approximately 2.5 to 15 µm^2^ (Figure 1, Figure 2, Figure 3, Figure 4 and Figure 5 and Figure 7, Table 1) with an average of approximately 5 µm throughout all layers investigated, although with a skewness to smaller SBs, as demonstrated by both TEM and FIB-SEM imaging. Beside similarities, striking differences in some structural parameters occurred when compared with their counterparts in experimental animals [31,32]. As in rodents and non-human primates, so-called *en passant* and end terminal SBs contacted either dendritic shafts (Figure 1A,B, Figure 2A, Figure 3C, Figure 4A and Figure 5A), but the vast majority (approximately 90%) of excitatory SBs was established on dendritic spines of different types including mushroom (Figure 2D, Figure 3B, Figure 4D, Figure 5B,C and Figure 7A,C), stubby (Figure 2B, Figure 4C and Figure 6D), filopodial (Figure 2C), elongated (not shown), and non-classifiable spines (Figure 1B, Figure 2B, Figure 3A,B and Figure 4B). Secondly, the majority of spines (approximately 80–90%) contained a so-called spine apparatus (Figure 2C,D, Figure 3B,C and Figure 4C,D), which is a derivative of the endoplasmic reticulum that is responsible for spine motility and stabilization of the synaptic complex during single or repetitive high-frequency stimulation. Thus, it was hypothesized that spines containing this structural sub-element partially contribute to the modulation of short-term synaptic plasticity [33].

Interestingly, so-called dendro-dendritic synapses, regarded as a feature of the developmental brain, seems to appear more frequently in the human TLN, most prominently in L2 and L3 when compared to the neocortex of experimental animals as revealed by qualitative inspection. In addition, so-called clathrin-coated pits were frequently observed in SBs of the human TLN, some of which are located near the AZ, suggesting a role in membrane trafficking. Clathrin-coated vesicles selectively sort cargo at the cell membrane, trans-Golgi network, and endosomal compartments for multiple membrane traffic pathways, for example exo- and endocytosis. A sub-population is used in SV formation at the AZ. Interestingly, several SBs contained so-called tangles (Figure 4C), or distortions of internal organelles as a possible sign for aging or degenerating terminals. The majority of SBs in the human TLN (approximately 85%) contained up to eight mitochondria of different shapes and sizes (Figure 2, Figure 3, Figure 4 and Figure 5 and Figure 7B,C) occupying approximately 10–20% of the total volume of individual SBs, which is comparable to numbers estimated from experimental animals. However, a layer-specific difference in mitochondrial content and correlation with bouton size was observed. Mitochondria were always associated with SVs and transfer SVs from the resting to the RP and RRP [34,35]. Thus, mitochondria in the human TLN, beside various other functions in nerve terminals, partially contribute to the refilling and thus replenishment of the RRP and RP of SVs.

Finally, as previously shown in experimental animals, astrocytes form a dense network throughout all layers of the human TLN. Synaptic complexes were tightly ensheathed by fine astrocytic processes, forming the “tripartite” synaptic complex (Figure 7A3) and separating them from adjacent synaptic complexes in the neuropil. Fine astrocytic processes could be followed as far as to the synaptic cleft under the pre- and postsynaptic densities. Hence, astrocytes can actively take up excessive or “spilled” neurotransmitters via glutamate transporters, thereby modulating the temporal and spatial neurotransmitter concentration in the synaptic cleft. Thus, astrocytes control the induction, maintenance, and termination of synaptic transmission but also modulate short-term synaptic plasticity in the human TLN. Strikingly, we could demonstrate that astrocytic processes receive direct synaptic input (Figure 3C, Figure 5A and Figure 6B), although infrequently, supporting their direct involvement in synaptic transmission and plasticity, for example beside various other functions controlling spike/time-dependent depression [36].

However, the most striking difference between SBs in the human, non-human primate, and rodent neocortex is the shape and size of the AZs and that of the three functionally defined pools of SVs, namely the RRP, RP, and resting pool (Table 1 and Table 2). Although comparably small in average size (see above), excitatory SBs in L1 to L6 of the human TLN contained AZs that were on average 2–2.5-fold larger in size (approximately 0.2–0.3 µm^2^ in surface area) when compared to SBs of comparable size in rodents or non-human primates or even much larger central nervous system (CNS) synapses (approximately 0.1 µm^2^) such as the cerebellar climbing fiber and mossy fiber boutons, hippocampal mossy fiber boutons terminating on spiny excrescences of apical dendrites of CA3 pyramidal neurons, and the calyx of Held-principal neuron synapse in the medial nucleus of the trapezoid body (Table 1 and Table 2).

In numerous (50–60%) SBs of the human TLN, the AZs covered most of the pre- and postsynaptic apposition zone at spines (Figure 2B,C, Figure 3A,B, and Figure 4B,D), hence enlarging the presynaptic “docking” zone for SVs. In addition, nearly one-third of the SBs contained not only a single but up to three AZs (Figure 2D, Figure 4C, and Figure 7B) in the human TLN. Beside macular, non-perforated AZs (Figure 2A,D and Figure 4A), also, perforated, ring- and horseshoe-like AZs (Figure 7A1) were found with a skewness to macular AZs. The size of the synaptic cleft was on average approximately 20–25 nm with slight differences between layers, but comparable with findings in experimental animals.

Even more strikingly, SBs in the human TLN contained a total pool of SVs (average 1500–1800 SVs, see also Figure 7; Table 2) that was in general 2 to 3-fold larger than that reported in the rodent and non-human primate neocortex. However, the total pool of SVs was highly variable in individual SBs (minimum 150; maximum 5000) similar to in experimental animals and showed layer-specific differences. These high numbers in the total pool suggested also comparably large RRPs, RPs, and resting pools. Indeed, using a perimeter analysis [24,25], the RRPs were on average 3 to 5-fold larger, the RPs are approximately 2 to 3-fold larger, and the resting pools are on average 2-fold larger than in the rodent and non-human primate neocortex. Using a perimeter (p) of 20 nm criterion, which is less than a vesicle’s diameter, the number of SVs in the RRP further increased by up to 4-fold when compared to experimental animals.

Thus, these large pools suggest reliable synaptic efficacy and strength even at high-frequency stimulation; hence, a rapid depletion of the RRP and RP could be prevented by the replenishment of SVs from a large resting pool. It has to be noted though that a huge variability exists in the structural composition between individual SBs and layers in the human TLN, in particular in the size of RRP, RP, and resting pool, which may partially contribute to modulating synaptic plasticity, but in a layer-specific manner. 

Taken together, the structural composition of both the presynaptic terminal and the spine as the main target structure together with the tight ensheathment of fine astrocytic processes suggested the high synaptic efficacy and reliability of synaptic transmission but also in the induction, regulation, and termination of short-term plasticity at synaptic boutons in the human TLN.

### 2.3. EM Tomography in the Human TLN

Technological advances in EM—for example, EM and Cryo-EM tomography—have opened a new door to image directly the sub-cellular and molecular organization of the pre- and postsynaptic density and promised new conceptual breakthroughs in the future. For example, the organization of the AZ and the pool of SVs, particularly those of the RRP, demonstrated a remarkable structural heterogeneity at the presynaptic density between individual SBs that allowed correlating structural heterogeneity with the functional characteristics of individual synapses as revealed by EM tomography [51,52,53]. 

However, to our knowledge EM tomography had never been performed on human tissue samples (Figure 6, Movie S2). Hence, we have used this approach to directly compare our perimeter measurements of the RRP (see above) by counting the number of “docked” (Figure 6A,A1,C, Movie S2) or already fused omega-shaped SVs (Figure 6D). In L4 of the TLN, the number of SVs within a perimeter of p10 nm was approximately 9 SVs, and there were approximately 20 SVs for the p20 nm criterion. The number of ‘docked’ or fused SVs using EM tomography was 5.5 SVs and thus approximately 2-4-fold smaller; however there was a large variability at individual AZs [25]. Preliminary results in other layers of the TLN lead to layer-specific differences in the RRP and RP with both EM tomography and perimeter measurements ranging from 2 to 8 SVs in the RRP. It has to be noted that the number of SVs in the RRP and RP was in general higher in all layers of the human TLN when compared with findings in the adult rat somatosensory cortex [31,32]. Strikingly, no or only a weak correlation between the size of SBs, the size of the presynaptic density, and the RRP and RP was found in both experimental animals and in the human TLN, pointing to an independent regulation of the RRP and RP at synaptic boutons in the human TLN. In summary, EM tomography verified and supported our perimeter analysis and appeared to be well suited for the estimation of vesicle pools, most importantly for that of the RRP in the human TLN.

## 3. Discussion

Here, we demonstrated that both TEM and FIB-SEM-processed tissue samples from neocortical non-epileptic access tissue obtained during epilepsy surgery was well-suited to quantitatively describe the synaptic organization of the human neocortex as exemplified by the TLN. Both experimental approaches allowed the estimation of structural parameters representing morphological correlates of synaptic transmission and plasticity that can be used to correlate structure with function directly. In addition, a direct comparison with findings from different animal species and brain regions where the quantitative data of synaptic structures are already available is now possible [31,32,37,38,39,47,54]. Furthermore, the quantitative 3D models of SBs could be used for numerical and/or Monte Carlo simulations of various synaptic parameters still not assessable for experiments, at least in humans.

### 3.1. Methodological Considerations

One possible way to describe SBs and their target structures in such great detail are either quantitative 3D volume reconstructions based on serial ultrathin sections using TEM imaging (Figure 1A, Figure 4 and Figure 5) or FIB-SEM (Figure 1B, Figure 2 and Figure 3, Movie S1). While for TEM, a series of consecutive ultrathin sections are examined while manual data acquisition is performed at the area of interest, such image stacks are generated inside the FIB-SEM by constant layer milling at the defined area of interest using a focused gallium ion beam in combination with an automated image acquisition right after every removed *z*-layer. From the resulting z-stacks of EM images using both EM techniques, quantitative 3D models of SBs and their prospective target structures could then be generated using different commercially or self-made reconstruction software tools running on high-performance computer systems (see Figure 6).

It should be mentioned that both imaging techniques, TEM as well as FIB-SEM, have advantages but also disadvantages. Serial sectioning and subsequent TEM examination of ultrathin sections within such a series is a very labor-intensive and thus a time-consuming process with a comparably low throughput of tissue samples. Table 3 summarizes the experimental time required to generate an aligned image stack from biopsy samples providing the basis for a subsequent 3D image reconstruction.

Secondly, in ultrathin sections, the tilting of the electron beam restricts the area of interest that could be investigated, and during the cutting and imaging process, malformations, distortions, or the complete loss of the ultrathin sections on a single slot grid can be a limiting factor. However, the major advantage of using serial ultrathin sections and subsequent TEM imaging is their very high quality reaching individual vesicle resolution. This is one requirement for the detailed analysis of important structural sub-elements such as the number, size, and shape of AZs and the organization and size of the three functionally defined pools of SVs that allow the generation of quantitative 3D models of synaptic structures.

In contrast, FIB-SEM, a relatively new, modern EM technology, allows a much higher throughput of tissue samples, because the time and labor-intensive step of serial ultrathin sectioning is no longer required. Secondly, a larger area of interest approximately 50 by 50 µm or even larger areas depending on the required resolution can be obtained compared to TEM where the area of interest is limited to approximately 10–25 by 10–25 µm, as it is restricted by the tilting of the EM beam. Finally, since the surface of the intact block is milled and polished, no malformations or distortions of individual images are expected, so only minor alignment processes of adjacent EM images should be required in the image post-processing phase. However, this expectation is often relativized to a certain extend as usually, a significant image shift is induced due to sample charging during image acquisition, which must be corrected either during the FIB-SEM run or also in the image post-processing. Beside the two major advantages of FIB-SEM, the speed in data acquisition and the possibility of investigating larger sample volumes defining regions of interest (ROIs) in one analysis run, this technique is rather challenging with respect to optimizing the quality of images. Concerning image quality, the first critical factor is the resolution potential of the underlying imaging system, FIB-SEM versus TEM. Regarding this parameter, one has to consider that an SEM has indeed a poorer resolution than a TEM of comparable technical standard. This could be mainly attributed to the standard sample thickness and the bulb-shaped electron beam interactions in deeper areas of the tissue sample. A modern SEM, similar to the Zeiss Crossbeam 540, has a maximum point resolution of about 1 nm under optimal conditions, whereas a comparable TEM reaches resolution limits of about 0.1 nm, which is a factor of 10× better. In practice, with the relatively thick resin-embedded samples, the best final resolution that we could obtain with our FIB-SEM was between 3 and 5 nm in the x–y dimensions. This was obviously worse than the resolution that we gained during the TEM analyses with 0.5 nm and constituted the first parameter that negatively influenced the overall image quality. Hence, the practical resolution of an FIB-SEM at present is not sufficient for a detailed study of vesicle fusion events at AZs. Even for the quantification and classification of SVs with a size of about 30–40 nm, a resolution of 5 nm should indeed be sufficiently enough to identify and discriminate SVs. However, due to a poorer resolution at appropriate higher magnifications, it is hard to separate individual SVs from each other that may result in either an over- or underestimation.

Nevertheless, the examples presented here of AZ and SV visualization by FIB-SEM did not only deliver a slightly poorer image result compared to the TEM data. At higher magnification, FIB-SEM images appeared significantly blurrier compared to corresponding TEM pictures, and as a result, it became harder to discriminate between individual SVs, impeding all subsequent quantification and classification approaches. It was obvious that not only the resolution potential of the respective microscope was crucial for the ultimate image quality. Using well-studied standard TEM sample preparation protocols, it is relatively easy to acquire TEM images with optimal contrast and good signal-to-noise ratios. Along the workflow for an FIB-SEM analysis, there are many more parameters that should be taken into account to optimize the image result. These options start with the general sample preparation procedure. In an SEM, the likelihood that scattering effects leading to secondary and back-scattered electrons leaving the sample in an upwards direction to contribute to the image formation is quite low, so that the overall contrast of an SEM image is generally poorer compared to a TEM picture. Consequently, the respective FIB-SEM users are constant searching for improved sample preparation protocols through which more electron dense molecules are deposited at defined ultrastructural elements, thereby increasing the structural contrast. In this study, we have applied a variation of a meanwhile very broadly used but also very complex 3D SEM sample preparation protocol developed by Mark Ellisman and co-workers [55]. Although delivering an already quite good contrast, it still offers space for adaptations to our unique samples. Ideas for optimizing the sample preparation include the use of conductive elements inside the embedding resin to increase the discharge capacity of the specimen [56]. This in turn would reduce charging effects, which contribute to sample instability during image acquisition. For the same reason, one has to optimize the thickness and composition of the sputter coat, and it could be meaningful to also address the overall sample thickness, which might have a negative effect on the thermic stability of the sample.

For image acquisition in an SEM, it is important to keep the accelerating voltage of the electron beam as low as possible, to induce the smallest bulb-shaped sample interaction of the electron beam and thereby receive the best possible point resolution. However, this parameter is critically dependent on the working distance and cannot fall below a certain threshold at a particular distance. In standard FIB-SEM systems, including our Zeiss system, this distance is relatively large due to construction reasons, but it has been shown by others that the system architecture might be optimized [57]. For current improvement approaches, it is important that the accelerating voltage of the FIB-SEM should be adjusted to minimal values to obtain the best possible resolution. A more flexible parameter is the electron beam current. Here, it is generally beneficial to use higher values to increase the signal strength released from the sample and thereby to optimize the signal-to-noise ratio. However, high beam current values inevitably lead to charging effects, which in turn cause sample instability during image acquisition, which ultimately leads to wiggly SEM pictures. Another parameter directly connected to the signal-to-noise ratio is the scan speed, which should be adjusted for every analysis. As for the beam current, a decrease in scan speed increases the beam pixel dwell time and therefore the resulting signal strength, but this immediately leads to charge depositions on the sample. A last image acquisition parameter that we would like to mention is the electron optics geometry. With our system, we can choose between different operation modes such as “analytical mode” and “high res mode”. These modes make use of the SEM’s double condenser system and preset the machine for different aims of analysis. However, while optimizing the condenser system for high-resolution imaging in “high res mode”, it also restricts the maximum beam current to 120 pA, which is a relatively low value and therefore limits the resulting signal strength. On the contrary, in “analytical mode”, the electron beam is not optimally focused on the sample surface, but higher beam current values are permitted. By naming just these most critical parameters, it is already obvious that setting up an FIB-SEM for 3D image acquisition always is connected to compromises between all individual machine parts, and that a broad experience is necessary to intuitively find the best possible definitions.

Regarding computational image post-processing, a critical factor directly impairing the 3D image quality was the anisotropic resolution of our FIB-SEM datasets with a voxel size of 5 nm × 5 nm × 50 nm [57]. So far, we have chosen the quite low z-resolution of 50 nm (1) to reduce the total image number and the image stack size to a minimum and (2) to generate comparable image stacks to our established serial sectioning TEM analyses. However, a switch to an isotropic resolution at a voxel size of 5 nm × 5 nm × 5 nm that still lies in the device specifications of our system might critically increase the 3D visualization quality of our FIB-SEM datasets and might also allow approaches of automated image segmentation by the use of artificial intelligence algorithms. However, both hypotheses remain to be elucidated in practice.

Apart from the blurry visualization of the target structures at high magnifications in the FIB-SEM, they also appeared structurally different from the TEM images. Potentially, this was due to the use of two individual buffer systems for the different sample preparation protocols (phosphate versus cacodylate buffer), but it is also very likely that the different EM embedding protocols had a substantial influence on the morphological appearance (see Figure 1B and Figure 4, Video S1).

In summary, there is still a plenitude of parameters in the context of FIB-SEM sample preparation and image acquisition that can be optimized to potentially fulfill our needs for a proper and reliable quantification and classification of the SV and AZ compartments in human brain biopsies. In combination with the much faster data acquisition speed and the option to analyze larger sample volumes, FIB-SEM would then be clearly the method of choice to answer most of our scientific questions. However, although working for 3 years with varying intensity on this project, we have not been able yet to define these optimal parameters, still making TEM analysis of serial sections the superior technique in our hands. Therefore, we believe that in the future, the combination of both TEM and FIB-SEM and further developments in fixation and embedding protocols will represent a comprehensive toolbox that will be inevitably needed to address all specific questions regarding the quantitative geometry of synaptic complexes and to further unravel the “microcosm” of the brain at the cellular and subcellular level. Here, the description of the “connectomics” and the synaptic organization of various layers, nuclei, and regions of the human brain will be of particular interest.

### 3.2. Functional Significance Working with Human Tissue Samples

As already mentioned above, one of the major questions in synaptic neuroscience is whether findings generated from experimental animals can be one-to-one transferred to the human brain. In the last two decades, it became possible to introduce structural and functional investigations also to the human brain, particularly the temporal lobe neocortex [14,15,16,17,18,19,24,25] by the use of accessing brain tissue taken from tumor or epilepsy surgery. It has been demonstrated that the structural heterogeneity of SBs in the human TLN is reflected in the functional properties of synaptic connections using paired recordings [14,15,16,17]. Excitatory synaptic connections in the human TLN are indeed highly reliable and strong, as indicated by large excitatory postsynaptic potentials (EPSPs) when compared to mouse neocortex, but also show layer-specific differences and in modulating short-term plasticity [17].

Hence, paired recordings of synaptic connections or directly from synapses and their target structures made it possible to measure transmitter release under defined internal and external ionic and membrane potential conditions. In addition, the size and time course of action potential evoked Ca^2+^ influx [58,59], the occupancy of the putative Ca^2+^ sensor driving vesicle fusion [60], the equilibration of intracellular Ca^2+^ with the endogenous Ca^2+^ buffer, and the eventual Ca^2+^ clearance [61] can be accurately measured. Furthermore, the latency, size, and time course of evoked quantal and multiquantal excitatory postsynaptic currents [14,17,30,32,62,63] can be determined. However, there are still steps in the signal cascades that at present could only be simulated [64,65]. This includes the site, time-, and space-dependent build up and collapse of Ca^2+^ domains around the pore of Ca^2+^ channels at a synaptic contact and the buffered diffusion and the subsequent interaction of free Ca^2+^with the Ca^2+^ sensor.

Thus, realistic values of the geometry of synaptic boutons, including the number, size, and shape of AZs, and the three functionally defined pools of SVs [62,63,66], namely the RRP, the RP, and the resting pool are essential for constraining the realistic geometrical models of synaptic structures. Thus, in the future, more structural or functional studies or correlated studies are required to describe the neuronal and synaptic organization of the human brain in more and sufficient detail.

### 3.3. Perspective to Work with Human Tissue Samples

As already mentioned above, neocortical non-affected access tissue from the TLN can be used to describe the neuronal and synaptic organization, for example of a cortical column, layer by layer. Meanwhile, numerous studies have shown that neocortical access tissue can, under the circumstances, be regarded as “normal” non-affected tissue, if the tissue samples were taken far from the epileptic focus or tumor as monitored by electroencephalography, direct electrophysiology, and magnetic resonance imaging before the operation. These investigations provide the basis to compare “healthy” human brain tissue with that of seizure (epileptic-) or tumor-affected tissue. Since we have almost finished the analysis of the layer-specific synaptic organization of the human TLN, we have recently started to investigate “epileptic” neocortical tissue samples from drug-resistant patients who did not undergo epilepsy surgery for years. Preliminary results demonstrated that depending on the number of years without operation, the affected neocortex developed dramatic signs of degeneration in the neuronal and synaptic organization of the neuropil (Lübke and co-workers, personal observations). Using the same experimental approach as described above, we started to investigate “affected” neocortical tissue samples to look for whether the arrangement of synaptic complexes, synaptic targeting, or the quantitative morphology of SBs will undergo dramatic changes and how that would influence their function. This can be achieved by realistic numerical and/or Monte Carlo simulation based on quantitative 3D models of synaptic structures of various parameters of the signal cascades, for example Ca^2+^ dynamics, transmitter release, and diffusion via the synaptic cleft on the presynaptic site and receptor sensitization and de-sensitization post-synaptically, which underlie synaptic transmission and plasticity. 

Secondly, neurotransmitter receptors are key players in synaptic transmission and plasticity at the molecular level. Some efforts have been made to study various neurotransmitter receptor systems using receptor autoradiography [67,68] or combined MRI/PET imaging [69,70] in the human brain. However, these approaches could not provide information about the possible co-localization, layer-specific density, and distributions pattern of certain neurotransmitter receptors and their subunits at individual postsynaptic densities (PSDs) at the subcellular level. Using a combination of freeze-fracture replication and high-sensitive single to multiple post-immunogold labeling, we started to investigate the receptor density and distribution pattern of the two global players in excitatory synaptic transmission in the human neocortex, namely α-amino-3-hydroxy-5-methyl-4-isoxazolepropionic acid and *N*-Methyl-d-Aspartat receptors. Preliminary results in non-affected, non-epileptic neocortical tissue samples demonstrated that both receptors and their subunits are co-localized but differentially expressed at individual PSDs located on dendritic shafts or spines, but with a huge variability in their density and distribution pattern (Lübke and co-workers, work in progress). From the normalized data, we will then generate receptor density maps of the distribution and overlap of both receptors at cortical synapses. To date, no such data exists for tumor-induced or epileptic tissue, which will be another step in our own investigations.

Hence, much more and extensive work is needed to unravel the neuronal, synaptic, and molecular organization of the human brain. 

## 4. Materials and Methods

### 4.1. Human Neocortical Tissue Processing for TEM, FIB-SEM, and EM Tomography 

Biopsy material was obtained from both male and female patients (25–63 years in age) who suffered from drug-resistant temporal lobe epilepsy and underwent surgery to control their seizures. The consent of the patients was obtained, and all experimental procedures were approved by the ethical committees of the Rheinische Friedrich-Wilhelms-University/University Hospital Bonn (ethic vote of the Medical Faculty to Prof. Dr. med. Johannes Schramm and Prof. Dr. rer. nat. Joachim Lübke, Nr. 146/11), and the University of Bochum (ethic vote of the Medical Faculty to PD Dr. med. Marec von Lehe and Prof. Dr. rer. nat. Joachim Lübke, Reg. No. 5190-14-15; ethic vote of the Medical Faculty to Dr. med. Dorothea Miller and Prof. Dr. rer. nat. Joachim Lübke, Reg. No. 17-6199-BR), and the EU directive (2015/565/EC and 2015/566/EC) concerning working with human tissue.

During surgery, blocks of neocortical access tissue from different regions of the superior, medial, and inferior temporal gyrus were taken far from the epileptic focus and may thus be regarded as non-affected (non-epileptic) tissue as routinely monitored by preoperative electrophysiology and magnetic resonance imaging. Other evidence confirming the “normality” of biopsy tissue samples rules out the effect of disease and treatment is the homogeneity of synaptic parameters analyzed among patients, as shown by [24,25]. The “normality” of human biopsy samples from epilepsy or tumor surgery has also been demonstrated by other recent structural and functional studies using the same experimental approach [15,17,19,71].

### 4.2. Fixation and Sectioning

After their removal from the brain, biopsy samples of the gyrus temporalis superior, medialis, and inferior of the TLN and sometimes hippocampus were immediately immersion-fixed in ice-cold, phoshate-buffered or cacodylate-buffered (0.1 M PB or CB, pH 7.4) 4% paraformaldehyde and 2.5% glutaraldehyde at 4 °C. After 4 h, the fixative was replaced by the same volume of the same freshly prepared fixative and stored for 24–48 h at 4 °C. 

Then, tissue samples were embedded in 4% agar–agar dissolved in PB and after hardening trimmed to remove the excess of agar–agar. Then, vibratome sections (150–200 µm in thickness, VT1000S, Leica Microsystems GmbH, Wetzlar, Germany) were cut in the frontal (coronal) plane through the TLN and collected either in ice-cold 0.1 M PB (for TEM analyses) or in ice-cold 0.15 M CB + 2 mM CaCl_2_ (for FIB-SEM analyses). Subsequently, two different protocols were used to prepare the tissue samples for either TEM or FIB-SEM.

### 4.3. Processing for TEM 

After thorough washing in PB (3 × 10 min), sections were post-fixed for 30 to 60 min in 0.5% or 1% osmium tetroxide (Sigma, Munich, Germany) diluted in PB-buffered sucrose (300 mOsm, pH 7.4) at room temperature in the dark. After visual inspection and thorough washing in PB, sections were covered with round coverslips in 18-well plates to prevent distortions of the sections caused by the dehydration process. Then, they were dehydrated in a series of ethanol starting at 20% to 90% ethanol (15 min for each step), followed by 20 min in 95% ethanol and absolute ethanol (2 × 30 min). This was followed by a brief incubation in propylene oxide (twice 2 min; Fluka, Neu-Ulm, Germany). Then, sections were transferred into a mixture of propylene oxide and Durcupan™ resin (2:1, 1:1 for 1 h each; Fluka, Neu-Ulm, Germany) and stored overnight in pure resin. The next day, they were flat-embedded either on coated glass slides or between Acla foils in fresh Durcupan™, coverslipped, and polymerized at 60 °C for 2 days.

### 4.4. Processing for FIB-SEM

Tissue samples were fixated and cut as already described above. Vibratome sections were collected in 0.15 M CB + 2 mM CaCl_2_ in multi-well plates and thoroughly washed several times in the same buffer solution (5 × 3 min). Thereafter, sections were incubated in 1.5% potassium ferricyanide, 2% osmium tetroxide, and 2 mM CaCl_2_ diluted in CB for 1 h, on ice. This was followed by a treatment in 1% aqueous thiocarbohydrazide for 20 min at room temperature (RT) and another subsequent incubation for 30 min in 2% aqueous osmium tetroxide solution at RT. Then, sections were block contrasted with filtered aqueous 1% uranyl acetate overnight at 4 °C before they were contrasted with Walton’s lead aspartate staining (aqueous solution of 20 mM lead nitrate in 30 mM aspartic acid) for 30 min at 60 °C. In between all the incubation steps, sections were thoroughly washed with the pure next diluent. After washing with deionized H_2_O (5 × 3 min), the sections were dehydrated through an ascending series of ethanol dilutions on ice (30%, 50%, 70%, 90%, 100%; 5 min for each step, followed by 2 × 10 min 100% ethanol dried on a molecular sieve). Next, sections were transferred to pure propylene oxide (PO; 2 × 10 min) and then processed through a series of Durcupan H™: PO mixtures with ascending resin concentrations (1:4, 1:2, 4:1; 2 h, each and pure resin overnight, followed by again pure resin for 2 h). The final pure Durcupan H™ solution was prepared from 100 g of component A, 100 g of component B, 3 g of component C, and 3 g of component D. After resin infiltration, sections were flat-embedded either between Acla foils or between a silicon-coated glass slide, and an appropriate piece of overhead transparency before samples were polymerized at 60 °C for two days. 

### 4.5. Serial Ultrathin Sectioning, TEM Data Acquisition, and 3D Volume Reconstructions

In the polymerized neocortical tissue samples, a region of interest was selected by visual inspections and glued on a pre-polymerized resin block. After trimming, semithin sections were cut with a Leica Ultracut S ultramicrotome (Leica Microsystems, Vienna, Austria). After toluidine-blue counterstaining, the final region of interest was selected for serial ultrathin sectioning (55 ± 5 nm in thickness, silver to light gray interference contrast appearance), and sections were collected on formvar- or pioloform-coated slot copper grids (Plano, Wiesbaden, Germany). Individual series comprised 75–150 ultrathin sections to allow the reconstruction of SBs of different size terminating on individual dendritic segments or spines to allow a representative sample of SBs of different shape and size. SBs were judged to be complete if the axon give rise to an end terminal bouton or if the axon could be followed in both directions with a swelling, leading to an *en passant* bouton that can be unequivocally identified from the beginning to its end in a series of ultrathin sections. Prior to TEM examination, sections were stained with uranyl acetate or uranyl less (Sciences Services, Munich, Germany; 15–20 min) and lead citrate (3–5 min) using a standard protocol by [72].

For EM tomography, 200–300 nm thick sections were generated according to the above stated protocol. These sections were collected on pioloform-coated line grids (Plano, Wiesbaden, Germany).

### 4.6. TEM Data Acquisition

Synaptic boutons and their target structures neurons were photographed from a series of ultrathin sections at a primary magnification of 8000× (Zeiss Libra 120, Oberkochen, Germany) using a Proscan 2K digital camera and the Panorama Function of the SIS Analysis (Olympus Soft Imaging System, Münster, Germany) or Image SP (Fa. Tröndle, Mohrenweis, Germany) software. Data were stored in a database until further use. In addition, interesting details about the synaptic organization within different layers of the neocortical gray matter and gyri were photographed at different magnification for further documentation within figure illustrations (see Figure 1A, Figure 4 and Figure 5).

EM tomography was carried out on 200–300 nm thick sections cut from blocks prepared for ultrathin sectioning as described above to quantify synaptic vesicles (SVs) particularly belonging to the RRP. Sections were mounted on either pioloform-coated line grids and were counterstained with uranyl acetate and lead citrate following a slightly modified staining protocol [72]. Subsequently, sections were examined with a JEOL JEM 1400Plus, operating at 120 kV and equipped with a 4096x4096 pixels CMOS camera (TemCam-F416, TVIPS, Gauting, Germany). Tilt series were acquired automatically over an angular range of −60° to +60° at 1° increments using Serial EM; Ver. 3.58 [73]. Stack alignment and reconstruction by filtered backprojection were carried out using the software package iMOD, Ver. 4.9.7 [74]. During the alignment procedure, the stack was binned in the x–y dimension by 2 pixels to reduce the noise and to increase the contrast of the final reconstruction.

### 4.7. FIB-SEM Data Acquisition

Based on the initial macroscopic appearance of the vibratome sections, areas of interest were cut out of the resin embedded tissue sample using a 4 mm biopsy puncher or a razor blade before they were glued onto a pre-polymerized resin block, using freshly prepared Durcupan™. After overnight polymerization at 60 °C, the mounted sample was trimmed precisely to the region of interest. An excess of resin on top of the tissue was removed by using a histology diamond knife on an ultramicrotome (UC7, Leica Microsystems, Vienna, Austria), leading to a smoothly polished surface and providing an optimal basis for the following FIB-SEM preparation steps. Next, the trimmed sample was removed from the underlying resin block with a razor blade and then glued onto an SEM aluminum specimen stub using a two-component conductive silver epoxy (Silver Conductive Epoxy, Ted Pella, Redding, CA, USA). The epoxy was cured at room temperature overnight, sputter-coated with a platinum/palladium layer of 20 nm thickness, and finally transferred into the FIB-SEM (Crossbeam 540, Carl Zeiss, Oberkochen, Germany) for 3D analysis.

A coarse trench was milled with the FIB at 30 kV/65 nA, polishing of the surface was performed at 30 kV/15 nA, and fine milling for data acquisition was performed at 30 kV/7 nA. The cross-section surface was imaged with an electron energy of 2 keV and an electron beam current of 500 pA (“analytical setting” of the column electron optics) using an in-column energy-selective backscatter electron detector. The dwell time was 10 ms with a line average of 1. The pixel size in the *x*–*y* plane was 5 nm, and the slice thickness (z-direction) was 50 nm, yielding a voxel size of 5 nm × 5 nm × 50 nm. The image acquisition software Atlas 3D (Ver. 5.2.0.125, ZEISS, Oberkochen, Germany) allowed the automated collection of 3D-SEM datasets using automated correction algorithms for drift, focus, and astigmatism. A final alignment of the resulting image stack was carried out to the serial section alignment workflow of the software package iMOD, Ver. 4.9.7 [74].

For a direct comparison between the TEM and SEM images, single sections of the FIB-SEM run were acquired using high-resolution SEM parameters: in detail, an electron energy of 1.5 keV at a beam current of 111 pA in “high-resolution” setting of the column electron optics. The pixel sizes and dwell times where chosen to achieve the best resolution and signal-to-noise ratio in the resulting image.

### 4.8. 3D-Volume Reconstructions 

To generate quantitative 3D models of SBs and their target structures, all calculations were performed offline using an open source batch version of OpenCAR, which generates 3D volume reconstructions as well as space-delimited tables for each measurement that are readable by standard analysis software. For further details on 3D-volume reconstructions, see [24,25,34].

Digital images were imported into OpenCAR, stacked, and transformed linearly such that corresponding structures were aligned along all consecutive images comprising the 3D image stack. In such individual *z*-stacks, all structures of interest, SBs, and their target structures were defined by different colors and codes. After contouring throughout all images, 3D-volumetric reconstructions were performed from which surface and volume measurements were obtained. 

In addition, the surface areas of the pre- (PreAZ) and postsynaptic density (PSD) were measured. Per definition, the PreAz and PSD constituting the AZ are regions of densely, electron-dense, dark-appearing material, condensed at the pre- and postsynaptic apposition zone. The surface areas of the PreAZ and PSD were computed separately by first generating a 3D surface model of the SB. Then, the PreAZ was measured by extracting this area from the reconstructed presynaptic bouton membrane that was covered by this membrane specialization (i.e., where the contour line coincided with less than 30 nm distance from the presynaptic membrane). Hence, the length (l) of the PreAZ (l PreAZ) and the surface area (SA) of the PreAZ (SA PreAZ) are already known. The size of the PSD opposing the PreAZ was estimated under the following assumptions: (1) both membrane specializations, PreAZ and PSD, run parallel to each other at the pre- and postsynaptic apposition zone; (2) for both membrane specializations, a contour line was drawn determining their actual length (l PreAZ and l PSD). Hence, the surface area of the PSD (SA PSD) is estimated by the following equation:

SA PSD = SA Pre × l PSD/l PreAZ(1)

which is the perimeter ratio between the outlines of the PSD to that of the synaptic contact.

The synaptic cleft width was measured at the two lateral edges and the center of the pre- and postsynaptic densities on digital electron microscopic images using the SIS software. Only synapses in which the AZ was perpendicularly cut, and which showed the typical broadening of the synaptic cleft were included in the sample (*n* = 5 animals, *n* = 125 AZs). The two values for the lateral edges were averaged, and a mean ± SD was calculated for each animal. Finally, a total mean ± SD over all animals was given.

### 4.9. Analyzing Vesicle Distribution and Pool Sizes 

To obtain estimates for the size of SVs and distance distributions, each vesicle in an axonal bouton was marked, thereby measuring its diameter and the minimal distance between its center of gravity and the presynaptic density of an AZ. This distance diminished by one vesicle radius gave an estimate of the minimal 2D distance that a vesicle had to bridge before it “touched” the membrane specialization. As the minimal 2D distance for each vesicle to an AZ was measured, it was possible to define different pools of SVs for each AZ depending on the vesicle perimeter and to plot their distribution. Errors in estimates of vesicle numbers were not applied for the numbers of small clear vesicles reported in this study [41]. For large dense-core vesicles, double counts were excluded by careful examination of adjacent images and were only marked in the image where they were the largest.

### 4.10. Statistical Analysis

From the numerous 3D reconstructions and spreadsheets computed by OpenCAR, statistical summaries and graphs were generated automatically using special purpose functions written for the freely available statistics package R (R Development Core Team. R: A language and environment for statistical computing. R Foundation for Statistical Computing, Vienna, Austria. ISBN 3-900051-07-0, URL http://www.R-project.org, 2005). Additionally, the non-parametric Kruskal–Wallis H-test analysis with post-hoc U-tests was used (GraphPad InStat Software Inc., San Diego, CA, USA) to obtain the differences between the several structural parameters investigated.

## Figures and Tables

**Table 1:** Comparison of structural parameters of SBs in different regions or layers of the rodent, non-human primate, and human neocortex.

Species (Strain)	Mouse (C57/BL6)*		Mouse (C57/BL6)**	Mouse (C57/BL6)***		Rat (Wistar)		Monkey (Macaca mulatta)***		Human	
**Region**	L4_M1	L4_S1	L4_S1	L2-3_V1	L2-3_FC	L4_S1	L5_S1	L2-3_V1	L2-3_LPFC	L4_TLN	L5_TLN
**Age**	—	—	60–65 days	2–14 month		90–120 days		5–20 years		20–63 years	
**Synaptic boutons**											
Surface area (µm^2^)	—	—	4.67 ± 2.20	—	—	3.03 ± 0.71	8.19 ± 2.84	—	—	2.50 ± 1.78	6.09 ± 0.85
Volume (µm^3^)	0.17^(+)^/0.07^(−)^	0.31^(+)^/0.07^(−)^	0.46 ± 0.27	0.10 ± 0.01	0.08 ± 0.03	0.20 ± 0.07	0.38 ± 0.23	0.20 ± 0.04	0.30 ± 0.01	0.16 ± 0.16	0.63 ± 0.17
**Active zones**											
Number per bouton	1.3^(+)^/1.1^(−)^	2.1^(+)^/1.2^(−)^	1.6	—	—	1.06 ± 0.06	1.12 ± 0.09	—	—	1–3	1–2
PreAZ surface area (µm^2^)	—	—	—	—	—	0.18 ± 0.06	0.29 ± 0.19	—	—	0.13 ± 0.07	0.23 ± 0.05
PSD surface area (µm^2^)	0.06^(+)^/0.56^(−)^	0.04^(+)^/0.04^(−)^	0.21 ± 0.11	0.08 ± 0.01	0.07 ± 0.02	0.18 ± 0.06	0.31 ± 0.21	0.08 ± 0.01	0.11 ± 0.01	0.13 ± 0.07	0.28 ± 0.11
**Cleft width (nm)**											
Lateral	—	—	—	—	—	17.22 ± 1.50	15.52 ± 0.39	—	—	14.11 ± 0.69	17.24 ± 2.21
Central	—	—	—	—	—	30.22 ± 1.42	31.32 ± 1.81	—	—	16.47 ± 1.85	19.05 ± 2.72
**Synaptic vesicles (SVs)**											
Total number	4846^(+)^/4861^(−)^	5032^(+)^/5233^(−)^	740 ± 285	—	—	561.00 ± 108.00	811.47 ± 272.25	337± 23	555 ± 48	1820.64 ± 980.34	1518.52 ± 303.18
**Pool size of SVs in:**											
Putative RRP at p10 nm	—	—	—	—	—	1.97 ± 2.57	3.89 ± 3.35	—	—	20.20 ± 18.58	5.42 ± 4.09
Putative RRP at p20 nm	—	—	—	—	—	6.30 ± 6.40	11.55 ± 4.16	—	—	48.59 ± 39.02	15.21 ± 9.09
Putative RP 60–200 nm	—	—	—	—	—	130.16 ± 20.79	162.83 ± 56.37	—	—	382.10 ± 248.23	181.86 ± 24.20
Putative resting pool >200 nm	—	—	—	—	—	408.84 ± 100.04	599 ± 212.21	—	—	1251.82 ± 471.17	1264.05 ± 269.91

The various synaptic parameters were taken from: * [37], (+): VGluT2-labeled boutons, (−): Unlabeled VGluT2 boutons; ** [38]; *** [39,40]; Rat “Barrel Cortex”: L4 and L5, [31,32]; Human TLN: L4 and L5, [24,25]; Abbreviations: L, layer; TL, temporal lobe; M1, primary motor; S1, primary sensory; V1, primary visual; FC, frontal cortex; LPFC, lateral prefrontal cortex; p (perimeter); 10 nm and p20 nm is the distance of synaptic vesicles from the AZ.

**Table 2:** Comparison of structural features of central synapses.

	Surface Area of SBs [µm^2^]	Number of AZs	Surface Area of AZs [µm^2^]
Calyx of Held	2500	554/*155 (85-217)	0.10 ± 0.08/*0.06 ± 0.12
Cerebellar Mossy Fiber	69–200/**168–266	191–440/**113–176/**~300	0.04 ± 0.02
Cerebellar Climbing Fiber	—	67	0.14 ± 0.08
Hippocampal Mossy Fiber	150–1000	2–40	0.11 ± 0.07

Data are taken from the following: Calyx of Held: [41]; * [42,43]; Cerebellar Mossy Fiber: [44]; ** [45]; Cerebellar Climbing Fiber: [46]; Hippocampal Mossy Fiber: [47,48,49]; [50]: Estimated from fluctuation analysis.

**Table 3:** Tissue processing for TEM and FIB-SEM.

Experimental Procedure	Time	
	TEM	FIB-SEM
Sample preparation (fixation, vibratome sectioning, osmification, dehydration, embedding, polymerization	120 h	125 h
Serial sectioning	3–5 h *	not applicable
Sputter coating, sample transfer into SEM + sample stabilization, deposition of protective metal pad, trench milling, and polishing of acquisition plane	not applicable	5 h
Data acquisition (x–y–z dimension; number of x–y images per stack	1–3 weeks **	24–30 h **
Data post-processing (parameter adjustment, bright/contrast, stack alignment, potential cropping)	not applicable	1 h
Total time	approximately 245–485 h ≅ 10–20 days	approximately 160 h ≅ 7 days

This table summarizes the total time of all individual tissue processing steps required to generate an aligned image z-stack compared for TEM or FIB-SEM. * Data acquisition critically depends on the number of ultrathin sections/series; ** Data acquisition time critically depends on the size of the z-stack and size of the area of interest.

## Supplementary Materials

Supplementary materials can be found at https://www.mdpi.com/1422-0067/21/15/5558/s1.

## Abbreviations

AZ	Active zone
CB	Cacodylate buffer
EM	Electron microscopy
FIB-SEM	Focused ion beam scanning electron microscopy
L	Layer
MRI/PET	Magnetic resonance imaging/positron emission tomography
PB	Phosphate buffer
PO	Propylene oxide
RP	Recycling pool
RRP	Readily releasable pool
RT	Room temperature
SB	Synaptic bouton
SV	Synaptic vesicle
TEM	Transmission electron microscopy
TLN	Temporal lobe neocortex
