# Supplementary material for: Synaptic Organization of the Human Temporal Lobe Neocortex as Revealed by High-Resolution Transmission, Focused Ion Beam Scanning, and Electron Microscopic Tomography
Source: Int J Mol Sci. 2020 Aug 3;21(15):5558. doi: 10.3390/ijms21155558 (PMC7432700; doi:10.3390/ijms21155558)
Supplement: Supplementary file 1 [file ijms-21-05558-s001.zip › ijms-871252-supplementary/ijms-871252-captions for supplementary videos.docx]

**Movie S1: Serial imaging through L2 of the human TLN obtained with FIB-SEM.** Movie S1 demonstrates the strategy of the FIB-SEM analysis. After trench milling to expose the region of interest, a stack of consecutive digital EM images was acquired, some of which are shown in the ongoing movie sequence through the neuropil of L2 in the human TLN. For image stack acquisition, the FIB sectioning was set up to result in virtual sections of 50 nm thickness. The reconstructed dendrite (blue) and SBs shown in Figure 6A are superimposed in the movie.

**Movie S2: EM tomography of a synaptic complex in L1 of the human TLN.** Movie S2 shows a high magnification of a large mushroom spine receiving excitatory synaptic input from an end terminal bouton in L4 of the human TLN. Note the occurrence of a prominent spine apparatus, the three AZs, and the “docked” SVs at the Pre-AZ. Scale bar: 0.25 µm.
